# Characterization of Factors Involved in Localized Translation Near Mitochondria by Ribosome-Proximity Labeling

**DOI:** 10.3389/fcell.2019.00305

**Published:** 2019-11-26

**Authors:** Dikla Vardi-Oknin, Yoav Arava

**Affiliations:** ^1^Faculty of Biology, Technion – Israel Institute of Technology, Haifa, Israel; ^2^Program in Nanotechnology and Nanoscience, Technion – Israel Institute of Technology, Haifa, Israel

**Keywords:** mitochondria, ribosomes, localized translation, mRNA localization, ribosome-proximity labeling, Tom20, Tom70, CLUH

## Abstract

Mitochondria exert their many functions through a repertoire of hundreds of proteins. The vast majority of these proteins are encoded in the nuclear genome, translated in the cytosol and imported into the mitochondria. Current models, derived mainly from work in yeast, suggest that the translation of many of these proteins can occur in close vicinity to the mitochondria outer membrane by localized ribosomes. Here, we applied ribosome-proximity biotin labeling to address this possibility. A clear biotinylation of ribosomes by mitochondrial Tom20-BirA fusion protein was observed in a human cell line. Isolation of these ribosomes revealed their preferred association with mRNAs encoding mitochondrial proteins. Furthermore, knock down of the mitochondrial protein receptor Tom70 resulted in a decrease in ribosomes translating mRNAs encoding proteins predicted to be recognized by Tom70. Intriguingly, levels of ribosomes translating mRNAs encoding targets of Tom20 were increased. We also knocked down the RNA binding protein CLUH that is implicated in regulation of mRNA encoding mitochondrial proteins, and found an increase in association of CLUH targets with mitochondria-proximal ribosomes. This is consistent with a role for CLUH in maintaining mRNAs encoding mitochondrial proteins in the cytosol. Overall, these data shed light on factors that contribute to association of translating ribosomes with human mitochondria and may suggest a co-translational mode of protein import into this organelle.

## Introduction

Human mitochondria contain more than a thousand proteins that exert its diverse functions (Calvo et al., 2016). The vast majority of these proteins are encoded in the nuclear genome, translated in the cytoplasm and imported into mitochondria (Schmidt et al., 2010; Becker and Wagner, 2018). Almost all proteins are inserted through the translocase of the outer membrane (TOM complex) (Kang et al., 2018). The TOM complex is composed of a central channel (Tom40 protein) surrounded by several protein receptors (primarily Tom20 and Tom70). These receptors recognize incoming proteins through various proteins signals (Mitochondrial Targeting Sequences). Tom20 interacts primarily with hydrophobic surface of N-terminal amphipathic helices (Mokranjac and Neupert, 2009). Tom70 on the other hand utilize targeting sequences throughout the substrate protein, and interact with Hsp90 and Hsp70 chaperones that bring precursor proteins (Young et al., 2003; Fan and Young, 2011). Nevertheless, an overlap between the two in targets’ recognition was also reported (Ramage et al., 1993; Brix et al., 1997; Backes et al., 2018).

Several mechanisms appear to mediate the approach of proteins to the TOM complex, and consequently their import (Hansen and Herrmann, 2019). The most studied mechanism involves complete translation of the protein in the cytosol, and then, with assistance of chaperones that maintain it in an unfolded state, transfer to one of the protein receptors of the TOM complex (Neupert and Herrmann, 2007; Kang et al., 2018). An alternative model involves local translation of mitochondria-destined proteins near the outer membrane, followed by its co-translational insertion through the TOM complex (Ahmed and Fisher, 2009; Lesnik et al., 2015). Data supporting this model includes the observation of ribosomes and mRNAs encoding mitochondrial proteins near yeast mitochondria (Kellems and Butow, 1972, 1974; Kellems et al., 1974, 1975; Marc et al., 2002; Garcia et al., 2007, 2010; Gadir et al., 2011) and the involvement of a translational process in this localization (Eliyahu et al., 2010). Few protein factors were found to be involved in this co-translational targeting of the protein, including the RNA binding proteins Puf3 (Saint-Georges et al., 2008; Eliyahu et al., 2010). Importantly, various components of the TOM complex also appeared to support the localization of mRNA and ribosomes to mitochondria vicinity, presumably through interaction with the protein nascent chain (Eliyahu et al., 2010, 2012; Gadir et al., 2011; Lesnik et al., 2014; Gold et al., 2017).

While the aforementioned studies establish the molecular mechanisms of localized translation in mitochondria of yeast, evidence in higher eukaryotes is largely missing. Previous studies had identified mRNA encoding mitochondrial proteins associated with mitochondria from human cell lines (Matsumoto et al., 2012; Fazal et al., 2019), plants (Vincent et al., 2017), and zebrafish (Sabharwal et al., 2018), yet association with the actual protein synthesis machinery (i.e., ribosomes) was not demonstrated. Furthermore, the *trans* factors that are involved in this process are largely unknown. Clustered mitochondria homolog (CLUH) is a protein that is likely to be involved in this process. CLUH is conserved from *S. cerevisiae* (Clu1), *D. melanogaster* (Cluless), and mammalian cells (CLUH) with important role in mitochondria morphology and physiology (Fields et al., 1998; Sen et al., 2013; Schatton et al., 2017). Importantly, the yeast, fly and the mammalian homologs all appeared to bind mRNA (Sen et al., 2013; Gao et al., 2014), with high preference for those that encode mitochondria proteins (Gao et al., 2014). Interestingly, the protein appears mostly in the cytoplasm, with a fraction appears associated with the mitochondria outer membrane (Gao et al., 2014; Sen and Cox, 2016). While the outer membrane localized CLUH was suggested to serve as an anchor for translating ribosomes near mitochondria (Sen and Cox, 2016), the cytosolic protein has a role in stability and translation regulation of this subset of mRNAs (Schatton et al., 2017). Thus, CLUH is proposed to affect localized translation process at multiple stages.

Here, we addressed questions of ribosomal association with mammalian mitochondria using the proximity-specific ribosome labeling (Williams et al., 2014). The outer membrane protein Tom20 was fused to the enzyme BirA, which allowed specific biotinylation of AviTag-carrying proximal ribosomes. Isolation of these ribosomes by virtue of their biotin tag revealed that they preferentially translate mRNAs encoding mitochondrial proteins. Knocking down Tom70 receptor revealed a clear reduction in localized translation of Tom70 protein-targets and an unexpected increase in Tom20-targets translation. Furthermore, knock down of CLUH revealed an increase in localization of its targets, consistent with a role in balancing cytosolic and mitochondrial localized translation.

## Materials and Methods

### Cell Culture and Growth Conditions

HEK-293T1 cells expressing the ribosomal protein RPL10A fused to AviTag-TEV-HA were kindly provided by Jonathan S. Weissman lab (UCSF) (Jan et al., 2014). HEK-293T1 cells were cultured in DMEM supplemented with 10% Fetal Bovine Serum (FCS), 2% penicillin/streptomycin, and 2 mM l-Glutamine. For biotin induction experiments, cells were cultured in DMEM supplemented with 10% FCS that was depleted of biotin (by a 2.5 h incubation with Streptavidin-Sepharose beads). For transfection, cells were grown to 70% confluence in DMEM and transfected using Jetprime reagent (Polyplus 11415).

### Plasmid Construction

Human Tom20 ORF was amplified by PCR from human cDNA, using the following primers: TOM20 F 5′-GCTAGCATGGTGGGTCGGAACA-3′ and TOM20 R 5′-GGTACCTTCCACATCATCTTCA-3′. mVenus protein linked to BirA was amplified by PCR from the plasmid pJW1507 (Addgene #62361) using the following primers: mVenus F 5′-ATGTCTAAAGGTGAAGAAT-3′ and BirA R 5′-TTATTTTTCTGCACTAGCT-3′. The fragments were cloned into the mammalian expression vector pcDNA3.1(+) under the control of CMV promotor.

### Live-Cell Confocal Imaging

Cells were seeded at the density of 250,000 cells per well in six-well glass-bottom plates for 24 h. Cells were then transfected with 1 μg TOM20-mVenus-BirA plasmid and after another 24 h mitochondria were stained with 100 nM MitoTracker^®^ Red CMXRos (Thermo Fisher Scientific) for 30 min before confocal live-cell imaging. For siRNA experiments, cells were transfected with 100 nM siRNA 48 h before imaging. All images were captured using LSM 710 inverted confocal microscope (Zeiss) with a 63 × 1.40 NA oil objective lens.

### Biotin Induction and Ribosomes Isolation

HEK-293T1 cells were seeded in a medium depleted of biotin 24 h before transfection with 10 μg TOM20-mVenus-BirA plasmid per 100 mm plate and grown for another 24 h in biotin-depleted medium before harvest. For siRNA treatments, cells were grown to 50% confluence in biotin-containing media and transfected with siRNA against human Tom70 (Dharmacon M02124301) (Fan et al., 2011), human CLUH (Invitrogen 1299003) (Gao et al., 2014) or control irrelevant siRNA (Sense 5′-UUCUCCGAACGUGUCACGU-3′) (Dong et al., 2017) in a final concentration of 100 nM. At this stage, growth was shifted to a biotin-depleted medium. Twenty four hours after siRNA treatment cells were transfected with TOM20-mVenus-BirA plasmid and grown for another 24 h in biotin-depleted medium. Cells were then treated with 100 μg/mL CHX for 2 min at 37°C and then d-Biotin (Sigma 58855) was added to a final concentration of 50 μM for additional 20 min. Next, cells were washed with PBS containing 100 μg/mL CHX and lysed with lysis buffer (20 mM Tris pH 7.5, 150 mM NaCl, 5 mM MgCl_2_, 2% Triton X-100, 1 mM DTT) on ice for 5 min. Cell lysate was cleared by spinning at 3,000 × *g* for 15 min and the supernatant was immediately loaded on Zeba de-salt spin column (Thermo Fisher Scientific 89882). Biotinylated ribosomes were isolated from the total cell lysate using MyOne streptavidin C1 magnetic DynaBeads (Invitrogen 65001). Prior to binding, beads were washed and equilibrated according to the manufacturer’s instructions. The pulldown was done on a roller for 1 h at 4°C. Then, the supernatant was removed and beads were washed three times with high-salt wash buffer (20 mM Tris pH 8.0, 750 mM KCl, 5 mM MgCl2, 100 μg/mL CHX, 0.5 mM DTT, 0.1% Triton X-100) for 15 min at 4°C. Bound ribosomes were either eluted by addition of 10U TEV protease (Invitrogen 12575-015) for 1 h at room temperature for protein elution or by adding 1 ml of TRizol for RNA extraction.

### Western Blot Analysis

Lysates were run on 11% PAGE, transferred to cellulose nitrate membranes and blocked with 5% BSA. The following antibodies were used: Mouse monoclonal anti HA (Covance MMS-101R) diluted 1:1000, Chicken polyclonal anti GFP (to detect mVenus) (Aveslab GFP-1020) diluted 1:2000, rabbit polyclonal anti GAPDH (Abcam Ab181602) dil. 1:2000, mouse monoclonal anti ATP5a (Abcam Ab119688) dil. 1:1000, anti rabbit polyclonal CLUH (Aviva System Biology ARP70642_P050) dil. 1:500. Biotin was detected directly using Streptavidin HRP conjugated (Abcam Ab7403) dil. 1:5000.

### RNA Extraction and Quantitative RT-PCR

RNA was extracted from Total lysate and Elution samples using TRIzol reagent. RNA were reverse transcribed with Maxima First Strand cDNA Synthesis kit (Thermo Fisher Scientific) and RT-qPCRs were performed using SYBR green (Thermo Fisher Scientific) with primers listed in Table 1. The fold change was calculated using the formula 2^(–ΔΔCt)^.

**TABLE 1:** List of primers used for qPCR.

**Gene**	**Forward**	**Reverse**
CLUH	5′-GGTAGCGGGCACGGTACA-3′	5′-CATTGAGCACCCCAACAC-3′
TOM70	5′-ACTACGAGCTACCTTCTACCT-3′	5′-CATGCTGCCTCTTTTGATGAG-3′
β-actin	5′-TCCCTGGAGAAGAGCTACGA-3′	5′-AGCACTGTGTTGGCGTACAG-3′
ATP5b	5′-TTGGTCCTGAGACTTTGGGC-3′	5′-CCTCAGCATGAATGGGAGCA-3′
MDH2	5′-TGAAGAACAGCCCCTTGGTG-3′	5′-GGTCCGAGGTAGCCTTTCAC-3′
CI-30	5′-GATGAAGTGAAGCGGGTGGT-3′	5′-GGCGATAGACTGGGAAAGCC-3′
COX6c	5′-ATGGCTCCCGAAGTTTTGCC-3′	5′-CCCCAGGGATAGCACGAATG-3′
PiC	5′-AGGATGGTGTTCGTGGTTTG-3′	5′-TGTGCGCCAGAGATAAGTATTC-3′
ANT1	5′-AGGGTTTCAACGTCTCTGTC-3′	5′-GTCACACTCTGGGCAATCAT-3′
ADH5	5′-GGCTCATGAAGTTCGAATCAAG-	5′-ACTCCCTCACCAACACTTTC-3′
ATP5a1	5′-GATCCGCTGCCCAAACC-3′	5′-GCCAATTCCAGCTTCATGGT-3′
OGDH	5′-AAGACCAAAGCCGAACAGTTTTA	5′-CGCCTCTCTCTGGGCCTT-3′
OPA1	5′-CCCTTCATAGCCAGCGAAGA-3′	5′-GAGTGAGAAAACAGCAACTGAATC
GOT2	5′-CACATCACCGACCAAATTGG-3′	5′-AGCCGCTCCACCTGTTCA-3′
TOM20	5′-ACAGAAACTTGCCAAGGAG-3′	5′-CTACGCCCTTCTCATATTCACC-3′
18S rRNA	5′-GTAACCCGTTGAACCCCATT-3′	5′-CCATCCAATCGGTAGTAGGC-3′

## Results

### Mitochondria-Proximal Ribosomes Translate mRNAs Encoding Mitochondrial Proteins

**FIGURE 1:**
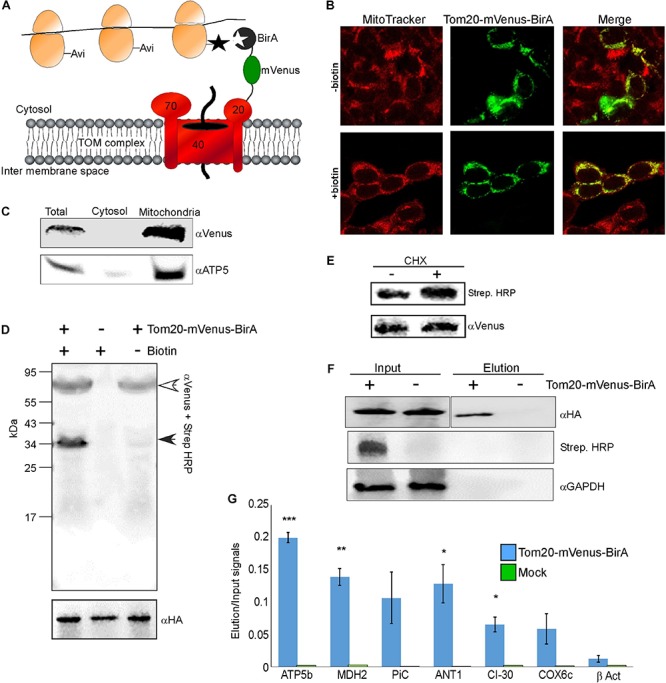
Identification of mRNAs translated near mitochondria. **(A)** Cells expressing HA-Rpl10A-AviTag were transfected with a plasmid expressing a fusion of Tom20-mVenus-BirA. Upon a biotin pulse, the BirA tags a proximal AviTag with a biotin. Biotinylated ribosomes are then isolated through a streptavidin column, and associated mRNAs are quantified by RT-qPCR. **(B)** Confocal microscope images of HEK-293T cells (either depleted or after re-addition of biotin) transfected with the Tom20-mVenus-BirA fusion, co-stained with the mitochondria marker MitoTracker Red CMXRos. **(C)** Fractionation analysis of cells expressing both HA-Rpl10A-AviTag and Tom20-mVenus-BirA. Samples were collected either before fractionation (Total) or after fractionation by differential centrifugation into cytosolic or mitochondrial fractions. Proteins were subjected to western analysis with antibodies recognizing mVenus and mitochondria marker (ATP5). Note that the mitochondria sample was five times more concentrated than the Total or cytosol samples. **(D)** Cells either expressing (+) or not (–) Tom20-mVenus-BirA were subjected to a pulse of biotin and immediately harvested. Samples were subjected to western analysis with Streptavidin-HRP and anti mVenus antibodies (simultaneously), and parallel samples were subjected to western analysis with anti HA antibodies. The open arrowhead indicates the signal of the Tom20-mVenus-BirA and the closed arrowhead the signal of the biotinylated HA-Rpl10A-AviTag. The anti HA indicate similar amounts of ribosomes in all lanes. **(E)** Cells expressing Tom20-mVenus-BirA were either treated for 2 min with cycloheximide (CHX) or not, and biotinylation levels were tested by western analysis. **(F)** Cells either expressing Tom20-mVenus-BirA (+) or not (–) were pulsed with biotin for 20 min and ribosomes were isolated through streptavidin beads. Ribosomes were eluted from the beads by cleavage with TEV protease (TEV site is present between Rpl10A and the AviTag). Samples from immediately after cell collection (Input) or from the eluted samples (Elution) were subjected to western analysis with the indicated antibodies. Note that the antiHA panels are from the same membrane from which irrelevant lanes were cut out. The apparent faster migration of the band in the Elution sample is due to the cleavage of the AviTag moiety by TEV during elution. **(G)** RNA was extracted from the Input and Elution samples and subjected to RT-qPCR with primers recognizing the indicated mRNAs. Histogram present the ratio of signals between the Elution and Input and are averages of two independent biological repeats each with three technical repeats. Error bars indicate the s.e.m. ^∗^*p* < 0.05, ^∗∗^*p* < 0.01, ^∗∗∗^*p* < 0.005, according to Student’s t-test with unpaired samples. Note that comparison of all samples was to the β-Act results.

We applied a proximity-specific ribosome tagging protocol (Williams et al., 2014) to tag mammalian mitochondria-proximal ribosomes. Biotin ligase (BirA) was fused to a mitochondrial outer membrane protein (Tom20) and a fluorescent protein (mVenus), and introduced into HEK-293T cells expressing HA-tagged ribosomal protein (HA-Rpl10A) fused to biotin acceptor (AviTag). Mitochondria-proximal cytosolic ribosomes are tagged by BirA upon a short pulse of biotin for cells grown in biotin-depleted media (Figure 1A). Fluorescent imaging revealed that the Tom20-Venus-BirA fusion protein is expressed at the mitochondria outer membrane (Figure 1B). Staining of mitochondria with the membrane-potential sensitive dye MitoTracker Red CMXRos (Figure 1B) revealed clear signal under depletion or addition of biotin, indicating functional mitochondria under these conditions. Cellular fractionation (Eliyahu et al., 2011) confirmed the absence of Tom20-mVenus-BirA from the cytosolic fraction (Figure 1C).

To demonstrate specificity of tagging, cells were subjected to biotin treatment and lysates were analyzed by western analysis using streptavidin-HRP probe. As can be seen in Figure 1D, only a single band, corresponding in size to the HA-Rpl10A-AviTag protein (∼35 kDa) is recognized. Negligible tagging is observed in cells that contain Tom20-mVenus-BirA yet were not pulsed with biotin. This small amount is probably due to residual biotin in the cells. Biotinylation in the presence of cycloheximide, which stalls ribosomes on mRNAs, was 70% higher compared to no treatment (Figure 1E). This pinpoints the correspondence between ribosomal association and tagging. Overall, this analysis confirms an efficient and specific biotinylation of Rpl10A-AviTag by the mitochondria-associated BirA.

Next, we performed proximity-specific ribosome isolation followed by RT-qPCR for candidate mRNAs. Cells, either expressing Tom20-mVenus-BirA or a mock control, were pulsed with biotin for 20 min, lysed and biotinylated ribosomes were pulled down by streptavidin beads. Ribosomes were eluted from the beads by cleavage with TEV protease (TEV site is present between the AviTag and HA-Rpl10A). Though lowers yield, this step further ensures specific isolation of target ribosomes. Western analysis revealed a signal for biotinylated protein only in the Input of the transfected cells, and not in the negative control cells. Furthermore, HA-Rpl10A signal was detected only in the Elution fraction of the Tom20-mVenus-BirA cells and not in the negative control (Figure 1F). Note that a biotinylation signal is absent from the Elution sample because the biotin moiety remains on the streptavidin beads due to the TEV-mediated elution.

RNA was extracted from the Input and the Elution samples, and mRNA was analyzed by real time quantitative PCR (RT-qPCR). We examined few mRNAs that encode mitochondrial proteins (ATP5b, MDH2, CI-30, COX6c, PiC, ANT1) and an mRNA that encodes a cytosolic protein (β-ACT). To account for differences in expression levels, signals from the Elution sample were normalized to the Input sample. Negligible signals are detected for these mRNAs in the mock treatment (Figure 1G), demonstrating low non-specific association with the beads. Importantly, mRNAs encoding mitochondrial proteins are enriched in the mitochondria-proximal ribosome fraction, much more than β-ACT mRNA. This is consistent with biotinylation occurring near the mitochondria and not randomly throughout the cytosol. Thus, mitochondria-proximal ribosomes are preferentially associated with mRNAs encoding mitochondrial proteins.

### Impact of Tom70 Knockdown

**FIGURE 2:**
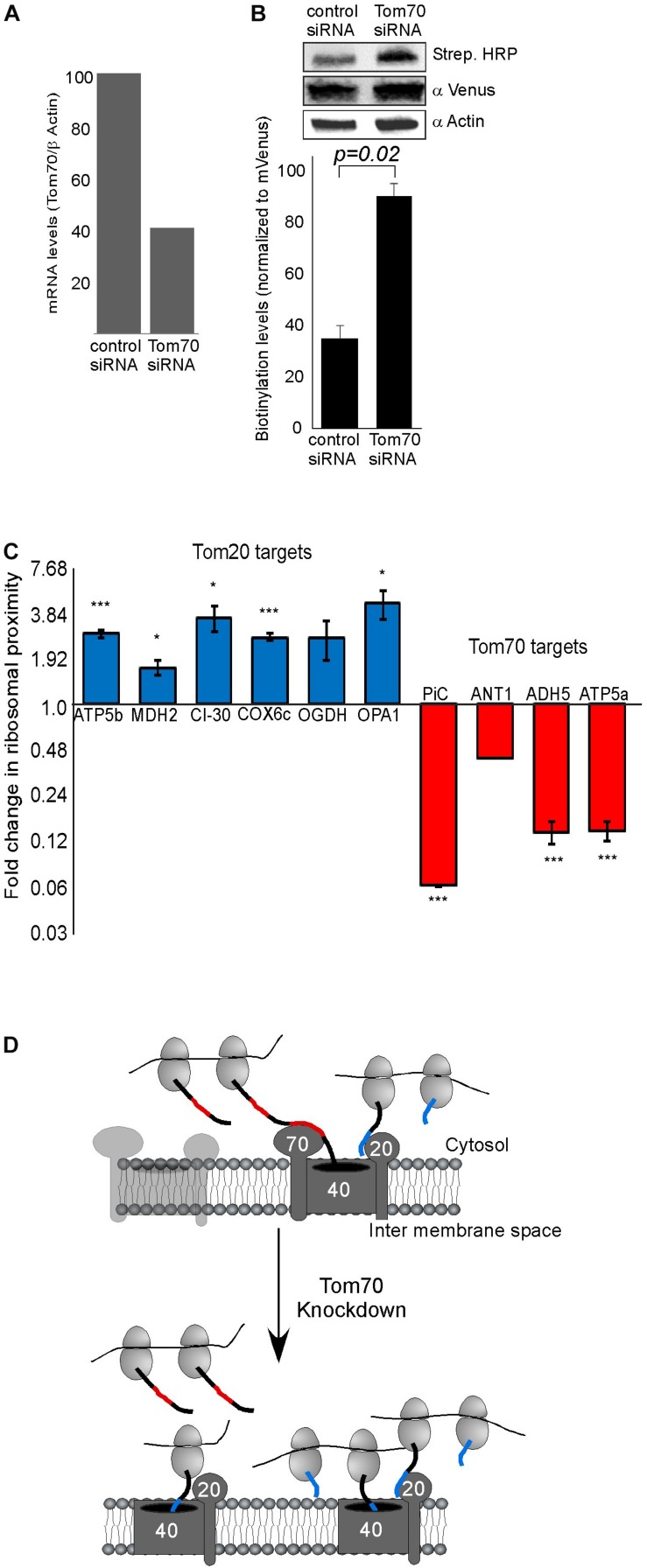
Tom70 impact on localized translation near mitochondria. **(A)** Cells were transfected with siRNA directed against Tom70 or control siRNA, harvested after 48 h and amounts of Tom70 mRNA was quantified by RT-qPCR. Signals are normalized to the levels of β-actin mRNA. **(B)** Cells either treated with siRNA for Tom70 or control were pulsed with biotin and harvested. Protein samples were subjected to western analysis with the indicated antibodies. Histogram presents the quantification of the Strep. HRP signal normalized to the mVenus signal from three independent biological repeats, and error bars are s.e.m. **(C)** Fold-change in Elution/Input ratio upon Tom70 depletion. Results are the average changes relative to the levels in the control siRNA treatment, from two independent biological repeats each with three technical repeats. Bars labeled blue indicate mRNAs encoding proteins with MTS that is predicted to be recognized by Tom20 and red are putative targets of Tom70. Data is presented in logarithmic scale for clarity. ^∗^*p* < 0.05, ^∗∗^*p* < 0.01, ^∗∗∗^*p* < 0.005, according to Student’s *t*-test with unpaired samples. Comparison of all samples was to no-change ratio (i.e., ratio of one). **(D)** Model for localized translation mediated by co-transport through Tom70 or Tom20. The interaction of targeting domains in the emerging nascent chain (depicted in Blue for Tom20-signals and Red for Tom70 signals) brings ribosomes to proximity with the mitochondria outer membrane. Upon Tom70 knockdown, ribosomes translating Tom70-targets are away from mitochondria while those translating Tom20-targets are enriched in proximity to the outer membrane.

Previous studies in yeast have implicated both Tom20 and Tom70 in association of mRNAs with mitochondria (Eliyahu et al., 2010, 2012). To examine this in human cells, we assayed the impact of Tom70 knockdown on ribosome biotinylation and mRNA association [Tom20 depletion appeared lethal to the cells (not shown)]. siRNA against Tom70 resulted in a significant reduction in Tom70 levels (Figure 2A). Biotin-labeling of ribosomes was performed and surprisingly biotinylation level was increased upon Tom70 knockdown (Figure 2B). Next, RNA samples were isolated from biotinylated ribosomes either from Tom70 knockdown or control cells, and levels of several mRNAs were tested (Figure 2C). We analyzed mRNAs that are predicted to express proteins that are imported in a Tom70-mediated manner (PiC, ANT1) (Söllner et al., 1990; Young et al., 2003), or proteins (ATP5b, MDH2, CI-30, COX6c) that carry a predicted N terminal MTS (Bannai et al., 2002) hence their import is likely Tom20-dependent. Down regulation of Tom70 resulted in a decrease in mRNAs encoding proteins that their import is Tom70-mediated (Figure 2C). Intriguingly, localization of mRNAs encoding proteins that are predicted to be targets of Tom20 increased in the Tom70 depleted cells. This may suggest a response mechanism in which Tom20-mediated import is increased in an attempt to compensate for Tom70 absence, and may account for the overall increase in biotinylation that is observed upon Tom70 depletion. Importantly, analysis for two mRNAs that lack a predictable N-terminal MTS (ADH5 and ATP5a), yet were never designated as Tom70 targets, revealed a change that resembles targets of Tom70 (i.e., decreased association upon Tom70 depletion). This suggests that the proteins encoded by ADH5 and ATP5a are imported in a Tom70-dependant manner. Furthermore, computational analysis of these proteins for the presence internal mitochondrial targeting sequences (Backes et al., 2018) suggested strong sites for ATP5a (data not shown), consistent with Tom70-mediated import. Taken together, we conclude that Tom20 and Tom70 receptors are involved in mitochondrial association of ribosomes that translate mitochondrial proteins (Figure 2D).

### CLUH Knock Down Impact on Localized Translation

**FIGURE 3:**
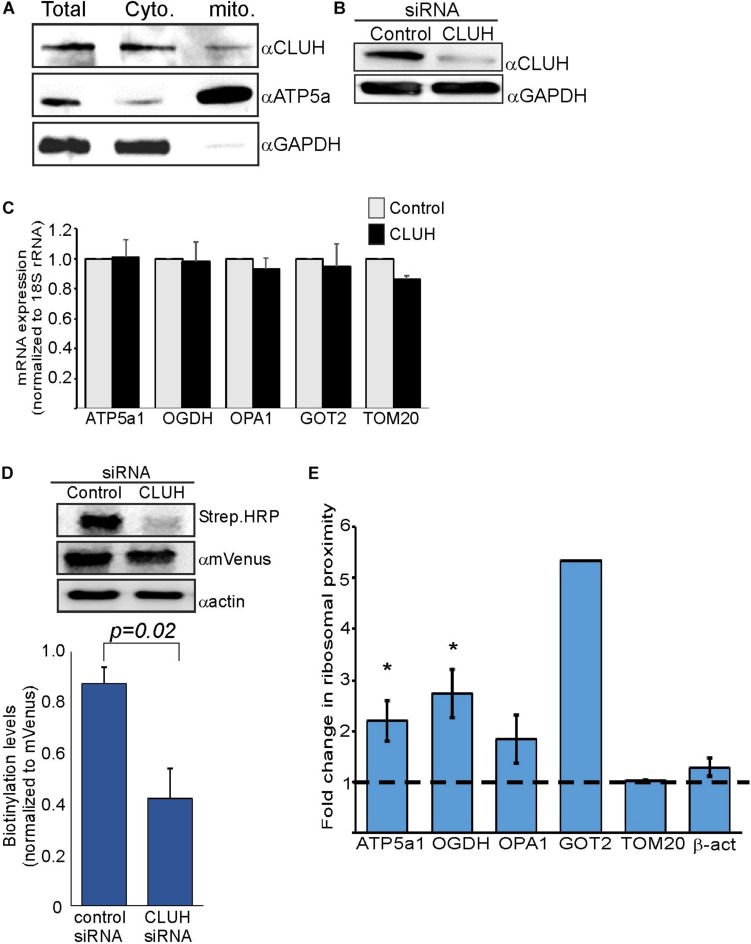
CLUH impact on localized translation near mitochondria **(A)** Cells were fractionated by differential centrifugation and sample before fractionation (Total) or after fractionation (Cyto. and Mito.) were subjected to western analysis with the indicated antibodies. Note that the mitochondria fraction is five times concentrated than the other samples. **(B)** HEK-293T1 cells were treated with siRNA toward CLUH or control siRNA, and protein samples were subjected to western analysis with the indicated antibodies. **(C–E)** Cells were transfected with control siRNA or CLUH and subjected to a biotin pulse. **(C)** RT-qPCR analysis for steady state mRNA levels of the indicated mRNAs. Error bars are s.e.m of two biological repeats each with three technical repeats. **(D)** Cells either treated with siRNA for CLUH or control were pulsed with biotin and harvested. Protein samples were subjected to western analysis with the indicated antibodies. Histogram presents the quantification of the Strep. HRP signals normalized to mVenus signals, from three independent biological repeats, and error bars are s.e.m. **(E)** Fold-change in Elution/Input ratio upon CLUH depletion. Results are the average changes relative to the levels in the control siRNA treatment from two independent biological repeats, each with three technical repeats. GOT2 data is from a single biological repeat. ^∗^*p* < 0.05, ^∗∗^*p* < 0.01, ^∗∗∗^*p* < 0.005, according to Student’s *t*-test with unpaired samples. Comparison of all samples was to no-change ratio (i.e., ratio of one).

CLUH is an RNA-binding protein (RBP) that preferentially interacts with mRNAs encoding mitochondrial proteins (Gao et al., 2014). It has a posttranscriptional regulatory role which affects the mitochondrial proteome and function (Schatton et al., 2017). We wished to investigate whether CLUH has a role in mitochondria localized translation of its target mRNAs. Cellular fractionation experiments (Eliyahu et al., 2011) revealed that the vast majority of CLUH is cytosolic and a small fraction is sedimenting with the fraction containing mitochondrial proteins (Figure 3A). Next, CLUH was knocked down (Figure 3B) to investigate its possible role in mitochondria localized translation. Steady-state mRNA expression of few mRNAs that encode mitochondrial proteins and were shown to be bound by CLUH (ATP5a1, OPA1, OGDH, GOT2) and a control mRNA that is not bound by CLUH (TOM20) (Gao et al., 2014) revealed small, if any, impact on their levels upon CLUH depletion (Figure 3C). This suggests that CLUH does not affect the stability of these mRNAs. Proximity labeling analysis upon CLUH depletion revealed a clear reduction in biotinylation (Figure 3D). Surprisingly, however, analysis of mRNAs associated with this lower amount of ribosomes revealed an increase in all CLUH-target mRNAs, but not for the control TOM20 or the non-mitochondria β-actin mRNA (Figure 3E). Thus, while the overall amount of ribosomes near mitochondria is decreased upon CLUH depletion, CLUH- targets exhibit a higher proximity to the mitochondria.

## Discussion

In this work, we provide support for localized translation of mRNAs encoding mitochondria-destined proteins. The proximity-labeling method is based on specific biotinylation of AviTag by the BirA enzyme. BirA biotinylates substrates that are in about 10 nm proximity, thus making this approach very efficient in detecting proximal interactions. We note that such distance does not allow determination of whether ribosomes are physically attached to the TOM complex (as is the case in co-translational import to the ER), or held in proximity by other means. Therefore, this data *per se* does not provide support to a co-translational import. Nevertheless, the impact of Tom70 depletion on mRNA localization may. We propose that the nascent protein chain interacts with Tom70 and thereby associates translating ribosomes with mitochondria (Figure 2D). This goes in line with previous work in yeast, that had shown the involvement of both Tom20 and Tom 70 receptors in co-translational import into mitochondria (Eliyahu et al., 2010, 2012). Interestingly, we see opposite changes in association of mRNAs predicted to be Tom70 targets compared to predicted Tom20 targets (Figure 2C). While mRNAs encoding Tom70 targets are reduced upon Tom70 depletion, Tom20-targets increase. It should be noted that in many cases the annotation of a protein as a Tom20 or Tom70-target is based on computational tools that may not always be accurate. For example, internal MTS-like sequence were shown to be involved in Tom70-mediated import (Backes et al., 2018). Yet, when we seek for such features in ADH5 and Atp5a1 (Backes et al., 2018), we identified four prominent ones in Atp5a1 and three weaker ones in ADH5 (data not shown). ADH5 therefore may have skipped identification as Tom70 target by this analysis. Thus, our experimental approach may serve as a tool to empirically determine the receptor of a protein; i.e., mRNAs with decreased association upon Tom70 depletion are likely to encode Tom70 targets, and mRNAs with increased association are likely Tom20 targets. Altogether, we suggest that co-translational import of mitochondrial proteins underlay the proximity of a subset of ribosomes that translate mRNAs encoding mitochondrial proteins (Figure 2D).

The RNA binding protein CLUH is known to preferentially bind mRNAs encoding mitochondrial proteins (Gao et al., 2014). We show that depletion of CLUH has a marginal effect on steady state mRNA levels of some of its targets. We therefore propose that CLUH has a post-transcriptional role, that is independent of mRNA stability. Most likely is translation regulation and mRNA transport to the mitochondria vicinity. Support to this possibility comes from the increase in amounts of CLUH-target mRNAs near the mitochondria (Figure 3E), concomitant with a decrease in the amount of ribosomes proximal to the mitochondria (as measured by biotinylation levels (Figure 3D). We therefore expand the working model proposed by Schatton et al. (2017), in which CLUH keeps its target mRNAs at the cytosol in a translationally active status. Hence, depletion leads to a decrease in their polysomal status [as was shown by Gao et al. (2014) and Schatton et al. (2017)]. We pose that the decreased polysomal status is accompanied with increased mitochondrial association, presumably to compensate for the lower protein synthesis rates.

Previous studies in diverse organisms revealed that many mRNAs are localized near mitochondria. The main novelty herein is that mRNAs are shown to be localized by virtue of their association with ribosomes. Thus, the mRNAs that we identified here are likely to be in the process of translation. Notably, our analyses were limited to few candidate mRNAs that were selected based on a prior knowledge. Unbiased approaches, presumably through the use of RNA-seq methodologies, are necessary to determine the extent of the phenomenon and the complete set of mRNAs that are translated near the mitochondria. We were unsuccessful in applying such methodologies thus far, and did not get any informative libraries from the Elution samples even with protocols that are aimed at nanogram amounts of mRNA. This is probably due to a combination of minute amounts of isolated mRNAs and its poor quality. Thus, higher scale preparation is probably necessary with optimization of the system to higher yields. Such analysis will significantly enhance our understanding regarding translation near the mitochondria outer membrane and the proteins that coordinate this process.

## Data Availability Statement

The datasets generated for this study are available on request to the corresponding author.

## Author Contributions

DV-O and YA designed the experiments, analyzed the results, and reviewed the final version of the manuscript. DV-O performed the experiments. YA wrote the manuscript and obtained the funding for this research.

## Conflict of Interest

The authors declare that the research was conducted in the absence of any commercial or financial relationships that could be construed as a potential conflict of interest.
